# 2-[(*E*)-(2-Phenyl-2*H*-1,2,3-triazol-4-yl)methyl­eneamino]ethanol

**DOI:** 10.1107/S1600536809007946

**Published:** 2009-03-11

**Authors:** Yan-Qiu Dang, Lai-Jin Tian

**Affiliations:** a Department of Chemistry and Chemical Engineering, Binzhou University, Binzhou 256600, People’s Republic of China; b Department of Chemistry, Qufu Normal University, Qufu 273165, People’s Republic of China

## Abstract

In the title Schiff base compound, C_11_H_12_N_4_O, the mol­ecule adopts a *trans* configuration about the central C=N bond. The dihedral angle between the phenyl ring and the triazole ring is 14.3 (3)°. In the crystal structure, mol­ecules are linked into a one-dimensional supra­molecular chain by inter­molecular O—H⋯N hydrogen bonding between the hydroxyl group and the imino N atom.

## Related literature

For related literature on Schiff bases, see: Ali *et al.* (2002 ▶); Borisova *et al.* (2007 ▶); Maheswari *et al.* (2006 ▶). For the crystal structures of similar Schiff bases, see: Nate *et al.* (1987 ▶); Yogavel *et al.* (2003 ▶). For standard bond-length data, see: Allen *et al.* (1987 ▶).
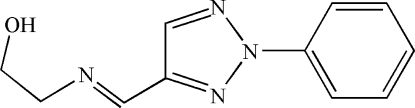


## Experimental

### 

#### Crystal data

C_11_H_12_N_4_O
*M* *_r_* = 216.25
Orthorhombic, 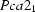

*a* = 13.124 (5) Å
*b* = 12.770 (5) Å
*c* = 6.658 (3) Å
*V* = 1115.8 (8) Å^3^
*Z* = 4
Mo *K*α radiation
μ = 0.09 mm^−1^
*T* = 295 K
0.12 × 0.07 × 0.03 mm

#### Data collection

Bruker SMART APEX area-detector diffractometer
Absorption correction: multi-scan (*SADABS*; Bruker, 2002 ▶) *T*_min_ = 0.989, *T*_max_ = 0.993
5534 measured reflections
1196 independent reflections
596 reflections with *I* > 2σ(*I*)
*R*_int_ = 0.084

#### Refinement

*R*[*F* ^2^ > 2σ(*F* ^2^)] = 0.057
*wR*(*F* ^2^) = 0.135
*S* = 1.01
1196 reflections
146 parameters
1 restraint
H-atom parameters constrained
Δρ_max_ = 0.15 e Å^−3^
Δρ_min_ = −0.14 e Å^−3^

### 

Data collection: *SMART* (Bruker, 2002 ▶); cell refinement: *SAINT* (Bruker, 2002 ▶); data reduction: *SAINT*; program(s) used to solve structure: *SHELXS97* (Sheldrick, 2008 ▶); program(s) used to refine structure: *SHELXL97* (Sheldrick, 2008 ▶); molecular graphics: *ORTEP-3 for Windows* (Farrugia, 1997 ▶); software used to prepare material for publication: *SHELXL97*.

## Supplementary Material

Crystal structure: contains datablocks global, I. DOI: 10.1107/S1600536809007946/wn2313sup1.cif

Structure factors: contains datablocks I. DOI: 10.1107/S1600536809007946/wn2313Isup2.hkl

Additional supplementary materials:  crystallographic information; 3D view; checkCIF report

## Figures and Tables

**Table 1:** Hydrogen-bond geometry (Å, °)

*D*—H⋯*A*	*D*—H	H⋯*A*	*D*⋯*A*	*D*—H⋯*A*
O1—H1⋯N4^i^	0.82	2.02	2.835 (6)	173

Symmetry code: (i) 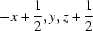.

**Fig. 1.**
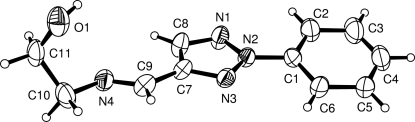
The molecular structure of the title compound, with displacement ellipsoids drawn at the 50% probability level. Hydrogen atoms are shown as spheres of arbitrary radius.

**Fig. 2.**
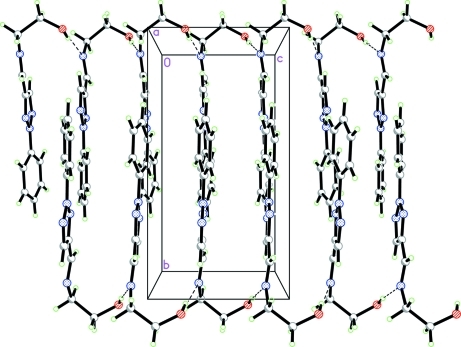
The packing of the title compound, viewed down the a axis. The intermolecular hydrogen bonds are shown as dashed lines.

## supplementary crystallographic information

### Comment

Schiff bases play an important role in coordination chemistry and have demonstrated significant biological activity (Ali *et al.*, 2002; Borisova *et al.*, 2007; Maheswari *et al.*, 2006). The title compound, derived from 2-phenyl-2*H*-1,2,3-triazole-4-carbaldehyde and 2-aminoethanol, is a potential N_3_O tetradentate Schiff base ligand. Its crystal structure is described here.

The title molecule (Fig. 1) adopts a *trans* configuration about the central C═N bond. The dihedral angle between the phenyl ring and the triazole ring is 14.3 (3)°. Atoms C9 and N4 are nearly coplanar with the triazole ring; the maximum deviation from the mean plane through these seven atoms is 0.066 (7) Å for C9. The torsion angles C8—C7—C9—N4 and N3—C7—C9—N4 are 8.4 (11) and -173.2 (6)°, respectively. The bond lengths (Allen *et al.*, 1987) and angles of the molecule are within normal ranges. The N4═C9 [1.255 (5) Å] and N4—C10 [1.461 (5) Å] bond distances are comparable to those found in similar Schiff base compounds, such as 2-(2-(2-(4-phenylpiperazinyl)ethoxy)benzylideneamino)ethanol (Nate *et al.*, 1987) and 1,4-bis(2-hydroxy-3-(*N*-(2-hydroxyethyl)imino)-5-methylbenzyl)piperazine (Yogavel *et al.*, 2003). In the crystal structure, O1—H1···N4 intermolecular hydrogen bonds (Table 1 and Fig. 2), formed between the hydroxyl group and the imino N, link the molecules into a one-dimension supramolecular chain.

### Experimental

2-Phenyl-2*H*-1,2,3-triazole-4-carbaldehyde (0.17 g, 1 mmol) and 2-aminoethanol (0.06 g, 1 mmol) were refluxed for 30 min in a methanol solution (15 ml). The reaction mixture was cooled to room temprature and filtered. After allowing the filtrate to stand in air for 3 d, pale yellow plate crystals (yield 76%; mp 346–347 K) were obtained.

### Refinement

H atoms were placed at calculated positions and refined in the riding-model approximation, with C—H = 0.93 Å and *U*_iso_(H) = 1.2U_eq_(C) for C*sp*^2^ H atoms, C—H = 0.97 Å and *U*_iso_(H) = 1.2U_eq_(C) for methylene H atoms, and O—H = 0.82 Å and *U*_iso_(H) = 1.5U_eq_(C) for the hydroxyl H atom. In the absence of significant anomalous scattering effects, Friedel pairs were merged.

### Crystal data

**Table:**

C_11_H_12_N_4_O	*F*(000) = 456
*M**_r_* = 216.25	*D*_x_ = 1.287 Mg m^−^^3^
Orthorhombic, *P**c**a*2_1_	Mo *K*α radiation, λ = 0.71073 Å
Hall symbol: P 2c -2ac	Cell parameters from 320 reflections
*a* = 13.124 (5) Å	θ = 2.2–18.5°
*b* = 12.770 (5) Å	µ = 0.09 mm^−^^1^
*c* = 6.658 (3) Å	*T* = 295 K
*V* = 1115.8 (8) Å^3^	Plate, pale yellow
*Z* = 4	0.12 × 0.07 × 0.03 mm

### Data collection

**Table:**

Bruker SMART APEX area-detector diffractometer	1196 independent reflections
Radiation source: fine-focus sealed tube	596 reflections with *I* > 2σ(*I*)
graphite	*R*_int_ = 0.084
φ and ω scans	θ_max_ = 26.0°, θ_min_ = 1.6°
Absorption correction: multi-scan (*SADABS*; Bruker, 2002)	*h* = −16→16
*T*_min_ = 0.989, *T*_max_ = 0.993	*k* = −13→15
5534 measured reflections	*l* = −7→8

### Refinement

**Table:**

Refinement on *F*^2^	Primary atom site location: structure-invariant direct methods
Least-squares matrix: full	Secondary atom site location: difference Fourier map
*R*[*F*^2^ > 2σ(*F*^2^)] = 0.057	Hydrogen site location: inferred from neighbouring sites
*wR*(*F*^2^) = 0.135	H-atom parameters constrained
*S* = 1.00	*w* = 1/[σ^2^(*F*_o_^2^) + (0.0327*P*)^2^] where *P* = (*F*_o_^2^ + 2*F*_c_^2^)/3
1196 reflections	(Δ/σ)_max_ < 0.001
146 parameters	Δρ_max_ = 0.15 e Å^−^^3^
1 restraint	Δρ_min_ = −0.14 e Å^−^^3^

### Special details

**Table:**

Geometry. All e.s.d.'s (except the e.s.d. in the dihedral angle between two l.s. planes) are estimated using the full covariance matrix. The cell e.s.d.'s are taken into account individually in the estimation of e.s.d.'s in distances, angles and torsion angles; correlations between e.s.d.'s in cell parameters are only used when they are defined by crystal symmetry. An approximate (isotropic) treatment of cell e.s.d.'s is used for estimating e.s.d.'s involving l.s. planes.
Refinement. Refinement of *F*^2^ against ALL reflections. The weighted *R*-factor *wR* and goodness of fit *S* are based on *F*^2^, conventional *R*-factors *R* are based on *F*, with *F* set to zero for negative *F*^2^. The threshold expression of *F*^2^ > σ(*F*^2^) is used only for calculating *R*-factors(gt) *etc*. and is not relevant to the choice of reflections for refinement. *R*-factors based on *F*^2^ are statistically about twice as large as those based on *F*, and *R*- factors based on ALL data will be even larger.

### Fractional atomic coordinates and isotropic or equivalent isotropic displacement parameters (Å^2^)

**Table:**

	*x*	*y*	*z*	*U*_iso_*/*U*_eq_	
N1	0.0897 (3)	0.2763 (3)	0.8805 (10)	0.0761 (13)	
N2	0.1661 (3)	0.3447 (3)	0.8868 (7)	0.0596 (11)	
N3	0.2589 (3)	0.3020 (3)	0.8840 (7)	0.0578 (10)	
N4	0.3009 (3)	0.0258 (3)	0.8507 (8)	0.0687 (13)	
O1	0.3829 (3)	−0.0715 (3)	1.2273 (8)	0.0842 (13)	
H1	0.3311	−0.0391	1.2570	0.126*	
C1	0.1492 (4)	0.4541 (4)	0.8940 (10)	0.0680 (14)	
C2	0.0539 (5)	0.4918 (4)	0.9368 (10)	0.084 (2)	
H2	0.0006	0.4456	0.9619	0.101*	
C3	0.0372 (6)	0.5982 (5)	0.9427 (11)	0.103 (3)	
H3	−0.0274	0.6241	0.9718	0.123*	
C4	0.1147 (7)	0.6647 (5)	0.9060 (11)	0.113 (3)	
H4	0.1035	0.7365	0.9139	0.136*	
C5	0.2086 (6)	0.6287 (4)	0.8579 (11)	0.107 (2)	
H5	0.2605	0.6758	0.8274	0.128*	
C6	0.2278 (5)	0.5209 (4)	0.8539 (9)	0.0877 (19)	
H6	0.2925	0.4954	0.8247	0.105*	
C7	0.2401 (3)	0.2000 (3)	0.8766 (9)	0.0602 (13)	
C8	0.1364 (3)	0.1845 (4)	0.8733 (11)	0.0720 (15)	
H8	0.1041	0.1197	0.8669	0.086*	
C9	0.3212 (4)	0.1209 (4)	0.8771 (10)	0.0673 (14)	
H9	0.3884	0.1414	0.8972	0.081*	
C10	0.3852 (3)	−0.0487 (4)	0.8635 (14)	0.0854 (19)	
H10A	0.4489	−0.0112	0.8791	0.103*	
H10B	0.3888	−0.0894	0.7408	0.103*	
C11	0.3691 (4)	−0.1209 (4)	1.0410 (13)	0.077 (2)	
H11A	0.3006	−0.1491	1.0349	0.093*	
H11B	0.4163	−0.1791	1.0308	0.093*	

### Atomic displacement parameters (Å^2^)

**Table:**

	*U*^11^	*U*^22^	*U*^33^	*U*^12^	*U*^13^	*U*^23^
N1	0.066 (2)	0.051 (2)	0.111 (4)	−0.004 (2)	0.003 (4)	0.007 (4)
N2	0.076 (3)	0.046 (2)	0.057 (3)	−0.001 (2)	0.002 (3)	0.005 (3)
N3	0.071 (2)	0.052 (2)	0.051 (3)	−0.0108 (19)	−0.002 (3)	0.001 (3)
N4	0.063 (2)	0.054 (2)	0.089 (4)	0.005 (2)	0.012 (3)	0.009 (3)
O1	0.070 (3)	0.066 (3)	0.117 (4)	0.0067 (19)	−0.014 (2)	−0.001 (3)
C1	0.100 (4)	0.052 (3)	0.053 (4)	−0.004 (3)	0.008 (4)	−0.006 (4)
C2	0.105 (4)	0.062 (4)	0.086 (6)	0.009 (3)	0.001 (4)	−0.005 (3)
C3	0.156 (7)	0.061 (4)	0.092 (6)	0.023 (4)	−0.005 (5)	0.002 (4)
C4	0.207 (9)	0.055 (4)	0.077 (6)	0.013 (5)	0.013 (7)	−0.010 (4)
C5	0.181 (8)	0.057 (4)	0.083 (6)	−0.028 (4)	0.043 (6)	−0.011 (4)
C6	0.132 (5)	0.056 (3)	0.075 (5)	−0.023 (3)	0.020 (5)	−0.004 (4)
C7	0.064 (3)	0.050 (3)	0.066 (4)	−0.008 (2)	−0.001 (4)	0.013 (3)
C8	0.068 (3)	0.047 (3)	0.101 (5)	0.000 (2)	−0.012 (5)	0.012 (4)
C9	0.063 (3)	0.066 (3)	0.073 (4)	−0.007 (2)	−0.004 (4)	0.014 (5)
C10	0.069 (3)	0.064 (3)	0.124 (6)	0.018 (3)	0.017 (5)	−0.011 (5)
C11	0.055 (3)	0.045 (3)	0.132 (7)	0.012 (3)	0.010 (4)	0.007 (5)

### Geometric parameters (Å, °)

**Table:**

N1—C8	1.324 (5)	C4—C5	1.354 (8)
N1—N2	1.330 (5)	C4—H4	0.9300
N2—N3	1.336 (5)	C5—C6	1.400 (7)
N2—C1	1.415 (6)	C5—H5	0.9300
N3—C7	1.327 (5)	C6—H6	0.9300
N4—C9	1.255 (5)	C7—C8	1.376 (6)
N4—C10	1.461 (5)	C7—C9	1.468 (6)
O1—C11	1.403 (8)	C8—H8	0.9300
O1—H1	0.8200	C9—H9	0.9300
C1—C6	1.365 (7)	C10—C11	1.514 (9)
C1—C2	1.371 (7)	C10—H10A	0.9700
C2—C3	1.377 (7)	C10—H10B	0.9700
C2—H2	0.9300	C11—H11A	0.9700
C3—C4	1.347 (9)	C11—H11B	0.9700
C3—H3	0.9300		
			
C8—N1—N2	103.5 (4)	C1—C6—H6	120.8
N1—N2—N3	114.8 (3)	C5—C6—H6	120.8
N1—N2—C1	122.1 (4)	N3—C7—C8	109.0 (4)
N3—N2—C1	123.1 (4)	N3—C7—C9	122.7 (4)
C7—N3—N2	103.4 (3)	C8—C7—C9	128.3 (4)
C9—N4—C10	117.4 (4)	N1—C8—C7	109.3 (4)
C11—O1—H1	109.5	N1—C8—H8	125.4
C6—C1—C2	120.8 (5)	C7—C8—H8	125.4
C6—C1—N2	119.5 (5)	N4—C9—C7	120.7 (4)
C2—C1—N2	119.7 (5)	N4—C9—H9	119.6
C1—C2—C3	119.8 (6)	C7—C9—H9	119.6
C1—C2—H2	120.1	N4—C10—C11	109.7 (5)
C3—C2—H2	120.1	N4—C10—H10A	109.7
C4—C3—C2	119.8 (7)	C11—C10—H10A	109.7
C4—C3—H3	120.1	N4—C10—H10B	109.7
C2—C3—H3	120.1	C11—C10—H10B	109.7
C3—C4—C5	121.1 (6)	H10A—C10—H10B	108.2
C3—C4—H4	119.4	O1—C11—C10	113.5 (5)
C5—C4—H4	119.4	O1—C11—H11A	108.9
C4—C5—C6	120.1 (6)	C10—C11—H11A	108.9
C4—C5—H5	119.9	O1—C11—H11B	108.9
C6—C5—H5	119.9	C10—C11—H11B	108.9
C1—C6—C5	118.3 (6)	H11A—C11—H11B	107.7
			
C8—N1—N2—N3	0.0 (8)	C2—C1—C6—C5	0.1 (10)
C8—N1—N2—C1	179.4 (6)	N2—C1—C6—C5	178.7 (6)
N1—N2—N3—C7	−0.3 (7)	C4—C5—C6—C1	1.8 (11)
C1—N2—N3—C7	−179.8 (5)	N2—N3—C7—C8	0.5 (7)
N1—N2—C1—C6	−165.0 (6)	N2—N3—C7—C9	−178.2 (5)
N3—N2—C1—C6	14.4 (9)	N2—N1—C8—C7	0.4 (8)
N1—N2—C1—C2	13.6 (10)	N3—C7—C8—N1	−0.6 (8)
N3—N2—C1—C2	−166.9 (6)	C9—C7—C8—N1	178.0 (6)
C6—C1—C2—C3	−1.0 (11)	C10—N4—C9—C7	−176.9 (6)
N2—C1—C2—C3	−179.6 (6)	N3—C7—C9—N4	−173.2 (6)
C1—C2—C3—C4	0.1 (11)	C8—C7—C9—N4	8.4 (11)
C2—C3—C4—C5	1.8 (12)	C9—N4—C10—C11	115.1 (7)
C3—C4—C5—C6	−2.8 (12)	N4—C10—C11—O1	−71.2 (6)

### Hydrogen-bond geometry (Å, °)

**Table:**

*D*—H···*A*	*D*—H	H···*A*	*D*···*A*	*D*—H···*A*
O1—H1···N4^i^	0.82	2.02	2.835 (6)	173

Symmetry codes: (i) −*x*+1/2, *y*, *z*+1/2.

## References

[bb1] Ali, M. A., Mirza, A. H., Butcher, R. J., Tarafder, M. T. H., Keat, T. B. & Ali, A. M. (2002). *J. Inorg. Biochem.* **92**, 141–148. 10.1016/s0162-0134(02)00559-7 12433421

[bb2] Allen, F. H., Kennard, O., Watson, D. G., Brammer, L., Orpen, A. G. & Taylor, R. (1987). *J. Chem. Soc. Perkin Trans. 2*, pp. S1–19.

[bb3] Borisova, N. E., Reshetova, M. D. & Ustynyuk, Y. A. (2007). *Chem. Rev.* **107**, 46–79. 10.1021/cr0683616 17212470

[bb4] Bruker (2002). *SADABS*, *SAINT* and *SMART* Bruker AXS Inc., Madison, Wisconsin, USA.

[bb5] Farrugia, L. J. (1997). *J. Appl. Cryst.* **30**, 565.

[bb6] Maheswari, P. U., Roy, S., Dulk, H., Barends, S., Wezel, G., Kozlevcar, B., Gamez, P. & Reedijk, J. (2006). *J. Am. Chem. Soc.* **128**, 710–711. 10.1021/ja056970+ 16417347

[bb7] Nate, H., Sekine, Y., Oda, K., Aoe, K., Nakai, H., Wada, H., Takeda, M., Yabana, H. & Nagao, T. (1987). *Chem. Pharm. Bull.* **35**, 3253–3260. 10.1248/cpb.35.3253 3427708

[bb8] Sheldrick, G. M. (2008). *Acta Cryst.* A**64**, 112–122. 10.1107/S0108767307043930 18156677

[bb9] Yogavel, M., Selvanayagam, S., Velmurugan, D., Shanmuga Sundara Raj, S., Fun, H.-K., Marappan, M. & Kandaswamy, M. (2003). *Acta Cryst. E* **59**, o83–o85.

